# Can health disparities be addressed by giving rural minorities dual consideration in the medical school admission process?

**DOI:** 10.1080/10872981.2023.2200549

**Published:** 2023-04-07

**Authors:** Forrest Bohler, Garrett W. Peters, Varna Taranikanti

**Affiliations:** School of Medicine, Oakland University William Beaumont, MS1, Auburn Hills, MI, USA; School of Medicine, Oakland University William Beaumont, MS2, Auburn Hills, MI, USA; Department of Foundational Medical Studies, Oakland University William Beaumont School of Medicine, Auburn Hills, MI, USA

Although the number of medical school positions has increased in recent years with the opening of new allopathic and osteopathic programs, issues surrounding the impending physician shortage persist. Physician recruitment and retention have become especially difficult in rural areas and are expected to increase in severity in the coming decades [1]. The lack of providers has resulted in poorer health outcomes for members of these communities. The recent COVID-19 pandemic has exacerbated these health disparities within rural populations, highlighting the need to address these inequities [2,3].

In recent years, there has been an increased focus on programs designed to promote physicians practicing in rural areas, ranging from rural medicine tracts within medical school curricula to loan repayment programs offered by state governments. Research has shown, however, that the greatest predictor of practicing in a rural community is dependent on whether the physician was from a rural area before matriculating into medical school [4]. As a result, medical schools have prioritized addressing this shortage in their admission process by giving special consideration to applicants from rural backgrounds.

Although these initiatives will be effective in addressing the shortage, in part, the issue of equity must be brought to light. On average, rural areas are predominantly white and have a lower percentage of minority groups than urban areas. In recent decades, however, this predominance has shifted as minority populations have been growing at increasing rates, with African Americans and Hispanics totaling 7.7% and 9.0% of the rural population, respectively [5]. Additionally, minority children under the age of 18 now comprise 32.5% of this age group population, indicating a further increase in the rural minority population in the near future [5].

While addressing the rural physician shortage is dependent on the number of practicing rural physicians, the quality of care that patients receive is dependent, in part, on the race of both the physician and patient. Studies have shown that patient outcomes are improved when physicians and patients share the same racial identity [6]. This paradigm highlights the need for the recruitment of rural minorities into medical school classes. Although Diversity, Equity, and Inclusion initiatives have been successful in increasing minority representation [7], we believe that dual consideration should be given to minority applicants hailing from rural backgrounds. This will ensure that rural health initiatives are equitably applied to benefit all members of these communities.
